# Argatroban in Patients With Acute Ischemic Stroke With Early Neurological Deterioration: A Randomized Clinical Trial

**DOI:** 10.1001/jamaneurol.2023.5093

**Published:** 2024-01-08

**Authors:** Xuting Zhang, Wansi Zhong, Rui Xue, Haidi Jin, Xiaoxian Gong, Yuhui Huang, Fujian Chen, Mozi Chen, Liqun Gu, Yebo Ge, Xiaodong Ma, Bifeng Zhong, Mengjie Wang, Haitao Hu, Zhicai Chen, Shenqiang Yan, Yi Chen, Xin Wang, Xiaoling Zhang, Dongjuan Xu, Yuping He, Minfang Lou, Aiju Wang, Xiong Zhang, Li Ma, Xiaodong Lu, Jianer Wang, Qiong Lou, Ping’an Qian, Guomin Xie, Xiaofen Zhu, Songbin He, Jin Hu, Xiongjie Wen, Yan Liu, Yanwen Wang, Jingjing Fu, Weinv Fan, David Liebeskind, Changzheng Yuan, Min Lou

**Affiliations:** 1 Department of Neurology, the Second Affiliated Hospital, Zhejiang University School of Medicine, Hangzhou, China; 2 School of Public Health, Zhejiang University, Hangzhou, China; 3 Department of Neurology, People’s Hospital of Anji, Huzhou, China; 4 Department of Neurology, First Hospital of Ninghai County, Ningbo, China; 5 Department of Neurology, The Affiliated People’s Hospital of Ningbo University, Ningbo, China; 6 Department of Neurology, Haiyan People’s Hospital, Jiaxing, China; 7 Department of Neurology, Putuo Hospital, Zhoushan, China; 8 Department of Neurology, Yiwu Central Hospital, Yiwu, China; 9 Department of Neurology, The Second Affiliated Hospital of Jiaxing University, Jiaxing, China; 10 Department of Neurology, Dongyang Affiliated Hospital of Wenzhou Medical University, Dongyang, China; 11 Department of Neurology, Zhuji People’s Hospital, Zhuji, China; 12 Department of Neurology, Quzhou Traditional Chinese Medicine Hospital, Quzhou, China; 13 Department of Neurology, Xiangshan People’s Hospital, Xiangshan, China; 14 Department of Neurology, Institute of Geriatric Neurology, The Second Affiliated Hospital and Yuying Children’s Hospital, Wenzhou Medical University, Wenzhou, China; 15 Department of Neurology, Shaoxing Second Hospital, Shaoxing, China; 16 Department of Neurology, The Affiliated Hospital of Hangzhou Normal University, Hangzhou, China; 17 Department of Neurology, The Second People’s Hospital of Yuhang District, Hangzhou, China; 18 Department of Neurology, The Affiliated Hospital of Medicine School, Ningbo University, Ningbo, China; 19 Department of Neurology, Ningbo Ninth Hospital, Ningbo, China; 20 Department of Neurology, Ningbo Medical Center Lihuili Hospital, Ningbo, China; 21 Department of Neurology, Quzhou City Kecheng District People’s Hospital, Quzhou, China; 22 Department of Neurology, Zhoushan Hospital, Wenzhou Medical University, Zhoushan, China; 23 Department of Neurology, Affiliated Hospital of Jiaxing University, Jiaxing, China; 24 Department of Neurology, Tongxiang Hospital of Traditional Chinese Medicine, Jiaxing, China; 25 Department of Neurology, Zhenhai Longsai Hospital of Ningbo city, Ningbo, China; 26 Department of Neurology, Zhejiang Hospital, Hangzhou, China; 27 Department of Neurology, The 4th Affiliated Hospital of Zhejiang University, School of Medicine, Yiwu, China; 28 Department of Neurology, Ningbo No.2 Hospital, Ningbo, China; 29 David Geffen School of Medicine, Department of Neurology and Comprehensive Stroke Center, University of California, Los Angeles; 30 Department of Nutrition, Harvard T.H. School of Public Health, Boston, Massachusetts

## Abstract

**Importance:**

The effect of argatroban in patients with acute ischemic stroke (AIS) and early neurological deterioration (END) is unknown.

**Objective:**

To assess the efficacy of argatroban for END in AIS.

**Design, Setting, and Participants:**

This open-label, blinded–end point, randomized clinical trial was conducted from April 4, 2020, through July 31, 2022. The date of final follow-up was October 31, 2022. This was a multicenter trial. Eligible patients were adults with AIS who experienced END, which was defined as an increase of 2 or more points on the National Institutes of Health Stroke Scale within 48 hours from symptom onset. Patients who withdrew consent, experienced duplicate randomization, or were lost to follow-up were excluded from the study.

**Interventions:**

Patients were randomly assigned to the argatroban group and control group within 48 hours of symptom onset. Both groups received standard therapy based on guidelines, including oral mono or dual antiplatelet therapy. The argatroban group received intravenous argatroban for 7 days (continuous infusion at a dose of 60 mg per day for 2 days, followed by 20 mg per day for 5 days) in addition to standard therapy.

**Main Outcome and Measure:**

The primary end point was good functional outcome at 90 days, defined as a modified Rankin Scale score of 0 to 3.

**Results:**

A total of 628 patients (mean [SD] age, 65 [11.9] years; 400 male [63.7%]) were included in this study (argatroban group, 314 [50%] and control group, 314 [50%]). Of these, 18 withdrew consent, 1 had duplicate randomization, and 8 were lost to follow-up. A total of 601 patients with stroke were included in the intention-to-treat analysis. Finally, 564 patients were included in the per-protocol analysis as 6 participants in the argatroban group and 31 participants in the control group did not follow the complete protocol. The number of patients with good functional outcome at 90 days was 240 (80.5%) in the argatroban group and 222 (73.3%) in the control group (risk difference, 7.2%; 95% CI, 0.6%-14.0%; risk ratio, 1.10; 95% CI, 1.01-1.20; *P* = .04). The proportion of symptomatic intracranial hemorrhage was 3 of 317 (0.9%) in the argatroban group and 2 of 272 (0.7%) in the control group (*P* = .78).

**Conclusions and Relevance:**

Among patients with AIS with END, treatment with argatroban and antiplatelet therapy resulted in a better functional outcome at 90 days. This trial provided evidence to support the use of argatroban in reducing disability for patients with END.

**Trial Registration:**

ClinicalTrials.gov Identifier: NCT04275180

## Key Points

**Question:**

Does argatroban improve neurological function in patients with acute ischemic stroke who experience early neurological deterioration?

**Findings:**

In this randomized clinical trial that included 628 patients with acute ischemic stroke and early neurological deterioration, good neurological function at 90 days in those randomized to receive argatroban plus antiplatelet compared with antiplatelet alone occurred in 80.5% vs 73.7% of participants, a difference that was statistically significant.

**Meaning:**

Among patients with acute ischemic stroke who experience early neurological deterioration, argatroban was significantly associated with better neurological function.

## Teaser

This randomized clinical trial investigates if argatroban improves neurological function in patients with acute ischemic stroke who experience early neurological deterioration.

## Introduction

Early neurological deterioration (END) within the first 48 hours after acute ischemic stroke (AIS) onset is relatively common, and it consistently predicts poor outcome.^1^ Apart from straightforward causes, such as intracerebral hemorrhage and malignant edema, the mechanism of END remains mostly unclear. Medical treatment in the setting of unexplained END in clinical practice may involve approaches such as plasma volume expansion, induced hypertension, and intensified antithrombotic therapy, but none has been formally proved so far.

Most patients with END still experience a significant worsening of their condition even with antiplatelet treatment.^2^ This may be attributed to the mixed potential etiologies, some of which are thrombus extension, hemodynamic compromise, and incomplete response to antiplatelet therapy. Theoretically, early use of anticoagulants, by reducing the propagation of a thrombus in an intracerebral artery, may reduce the volume of infarcted cerebral issue and subsequently decrease the risks of disability and death. However, it is noteworthy that anticoagulants also carry the risk of bleeding. Studies have showed that, although anticoagulants prevent stroke progression, the benefit of decreased recurrence by anticoagulants was offset by a similarly sized increase in intracranial hemorrhage.^3^ Early warfarin use in transient ischemic attack (TIA) or minor stroke was also associated with increased intracranial hemorrhage risk.^4^ Guidelines still recommend against urgent anticoagulation for patients with AIS.

As a direct thrombin inhibitor, argatroban is rapid acting, short acting, and has low bleeding rates,^5^ which may be useful in preventing thrombus propagation and providing additional benefit after stroke/TIA.^5^ Growing preclinical evidence has demonstrated that argatroban was associated with a reduction in ischemic stroke damage.^6,7^ However, the safety and efficacy of argatroban is not well established for AIS treatment, and there is also a lack of robust evidence for the effect of argatroban in patients with AIS who experience END.

We performed a prospective, multicenter, open-label, blinded–end point, randomized clinical trial aiming to explore the efficacy and safety of argatroban combined with antiplatelet therapy for END within 48 hours after symptom onset.

## Methods

### Study Design

The study protocol is available in Supplement 1, and the statistical analysis plan is available in Supplement 2. The trial was conducted at 28 medical sites in China. The trial protocol was approved by the appropriate regulatory and ethical authorities of the ethics committee of the Second Affiliated Hospital of Zhejiang University School of Medicine and other participating hospitals. An independent data monitoring committee monitored the progress of the trial every 6 months. Signed informed consent was obtained from patients or their legally authorized representatives. This study followed the Consolidated Standards of Reporting Trials (CONSORT) reporting statement.

### Participants

Eligible patients were adults older than 18 years with AIS within 48 hours and experienced END with an increase of 2 or more points on the total National Institutes of Health Stroke Scale (NIHSS). A full list of inclusion and exclusion criteria is available in the study protocol (Supplement 1). Information regarding participant race and ethnicity was not gathered for this study as most patients in Zhejiang Province were Han Chinese.

### Randomization and Masking

Patients were randomly assigned to either treatment or control arms using a secure, web-based randomization system. A dynamic stratification system ensures well-balanced subgroups. The randomization algorithm uses biased-coin minimization and the variance method with stratification weights. The strategy was to balance treatment assignment along the marginal distribution of each stratification factor. The stratification factors used and their hierarchy were as follows: (1) age, (2) sex, and (3) NIHSS score at randomization. Patients and treating physicians were not blinded to treatment assignment, but study outcomes assessors were blinded to treatment assignment.

### Procedures

Both groups received standard therapy including oral mono or dual antiplatelet therapy, such as aspirin and/or clopidogrel, decided by the attending physicians according to Chinese Stroke Association guidelines for diagnosis and treatment of acute ischemic stroke 2018.^8^ The experimental group received intravenous argatroban for 7 days (continuous infusion at a dose of 60 mg per day for 2 days, followed by 20 mg per day for 5 days) in addition to standard therapy. Argatroban infusion was terminated immediately if major systemic bleeding or symptomatic intracerebral hemorrhage was suspected. Patients had to be at least 24 hours postthrombolysis treatment and have no evidence of hemorrhage on computed tomography (CT) scans. The NIHSS was used to assess neurological status at baseline, 7 days, and 90 days after randomization. A detailed flowchart of the assessment schedule is provided in the study protocol (Supplement 1). Data on demographic and clinical characteristics were obtained at randomization. Follow-up data were collected at 7 days or at hospital discharge and 90 days after randomization. Remote and on-site quality control monitoring and data verification were conducted throughout the study.

### Outcome

The primary end point was good functional outcome at 90 days, defined as a score of 0 to 3 on the modified Rankin Scale (mRS) for the evaluation of neurological disability, through a structured interview for telephone assessment by personnel certified in the scoring of the mRS 90 days after randomization.

The secondary end points were favorable functional outcome (mRS score of 0-2) at 90 days, recovery assessed by categorical shift in mRS at 90 days, NIHSS score at 7 days and 90 ± 3 days after randomization, Barthel scale score at 90 ± 3 days, and the rate of composite cardiovascular events at 90 ± 3 days, including cerebrovascular events, myocardial infarction, angina pectoris, and systemic embolism.

Any adverse events that occurred during the study were recorded. The prespecified adverse event outcomes were symptomatic intracerebral hemorrhage, parenchymal hematoma type 2, and other common adverse events. Symptomatic intracranial hemorrhage was defined as any evidence of bleeding on head CT scan associated with clinically significant neurological deterioration (≥4 point increase in NIHSS score) in the opinion of the clinical investigator or independent safety monitor. Parenchymal hematoma type 2 was defined as confluent bleeding occupying more than 30% of the infarct volume and causing significant mass effect. Follow-up head CT was performed at 7 days after randomization or at any time when neurological deterioration occurred. Central adjudication of clinical outcomes and adverse events was done by assessors unaware of treatment allocation or clinical details.

Final follow-up was performed at 90 days through a structured interview for telephone assessment by a trained and certified member in the central research hospital who was unaware of the randomized treatment assignment. A training course was held for all the investigators at each center to ensure the validity and reproducibility of the evaluation, and only certified investigators were eligible to evaluate NIHSS scores.

### Sample Size Calculation

The sample size was estimated according to the results of the previous observational cohort (with the same inclusion and exclusion criteria as the randomized clinical trial) at the study center, with 33 patients in the experimental group and 66 patients in the control group. There were no significant differences in age and NIHSS score (baseline and progressive time), and the proportion of patients with an mRS score of 0 to 3 at day 90 in the experimental group and the control group was 80% and 69.6%, respectively. Based on 0.8 power to detect a significant difference (2-sided *P* = .05) and to compensate for nonevaluable patients of 15%, a total sample size of up to 630 patients would be required.

### Statistical Analysis

The modified intention-to-treat analyses were performed on the full analysis set, which included all patients who received at least 1 study protocol treatment and at least 1 efficacy evaluation after baseline. Generalized linear models (GLMs) were performed for the analyses of the primary and secondary outcomes of good functional outcome at 90 days, favorable functional outcome at 90 days, NIHSS score at 90 days, Barthel scale score at 90 days, and stroke or other vascular events within 90 days. The treatment effects for these outcomes are presented as risk ratios (RRs) and risk differences (RDs) with their 95% CIs. The mRS scores at 90 days were compared using ordinal logistic regression, and odds ratios (ORs) with 95% CIs were calculated. No interim analyses were performed in this study.

The primary analyses of the primary and secondary outcomes were unadjusted. Covariate-adjusted analyses were also performed for all outcomes, adjusting for 4 prespecified prognostic factors: age, sex, NIHSS score at randomization, and time from the index event symptoms to randomization. The missing values of baseline variables in the covariate-adjusted analyses were imputed using simple imputation methods based on their sample distributions.

A subgroup analysis of the primary outcome was performed on 5 prespecified subgroups: age (<65 years or ≥65 years), sex (female or male), NIHSS score (≤8 or >8), time from the onset of index event symptoms to randomization (≤24 hours or >24 hours), and reperfusion therapy (yes or no). Assessment of the homogeneity of the treatment effect by a subgroup variable was conducted using a GLM with the treatment, subgroup variable, and their interaction term as independent variables, and the *P* value for the interaction term was presented. Detailed statistical analyses are described in the statistical analysis plan (Supplement 2).

In addition, per-protocol analyses for primary and secondary outcomes were performed on patients who were treated appropriately according to the protocol. Analyses of adverse events were based on the safety population, which consisted of all randomized participants who received at least 1 dose of the argatroban. A 2-sided *P* value <.05 was considered statistically significant. Because of the potential for type I error due to multiple comparisons, findings for secondary outcome analyses should be interpreted as exploratory. SPSS software, version 23 (IBM), and R software, version 4.1.0 (R Foundation), were used for the statistical analyses.

## Results

### Trial Population

**Figure 1.**
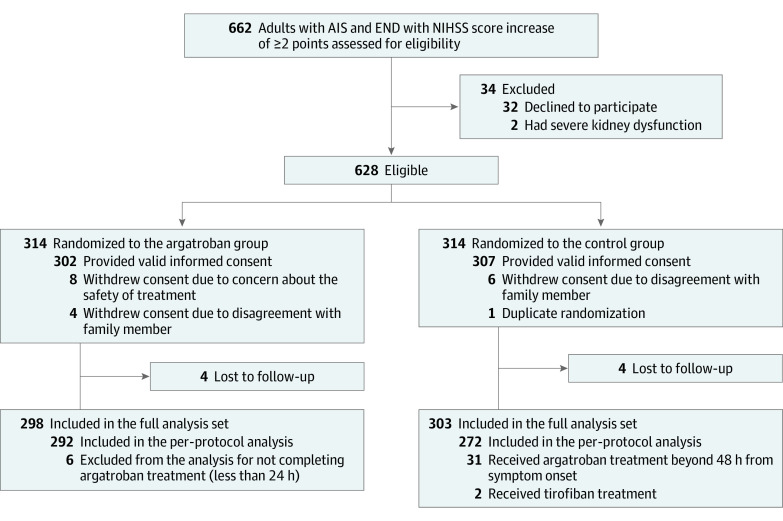
Patient Flow in Argatroban in Patients With Acute Ischemic Stroke With Early Neurological Deterioration: A Randomized Clinical Trial AIS indicates acute ischemic stroke; END, early neurological deterioration; NIHSS, National Institutes of Health Stroke Scale.

Between April 4, 2020, and July 31, 2022, a total of 628 patients (mean [SD] age, 65 [11.9] years; 400 male [63.7%]; 228 female [36.3%]) were randomly assigned to the argatroban group (314 [50%]) or the control group (314 [50%]). A total of 27 patients were excluded (18 withdrew consent, 1 had duplicate randomization, and 8 were lost to follow-up). The full analysis set population included 601 patients (298 [49.6%] in the argatroban group and 303 [50.4%] in the control group). The procedure was completed according to the protocol for 564 patients (292 [51.8%] in the argatroban group and 272 [48.2%] in the control group), and the results were included in the per-protocol analysis. The reasons for the incomplete procedures are provided in Figure 1. The trial was completed on October 31, 2022.

The treatment groups were well balanced with respect to baseline patient characteristics in the full analysis set population (Table 1) and per-protocol analysis set population (eTable 1 in Supplement 3). In the argatroban group, 292 of 298 patients (98.0%) underwent the complete procedure of argatroban at a median (IQR) of 24 (15-34) hours from symptom onset to randomization. The remaining 6 patients did not receive complete argatroban treatment. In the control group, 272 of 303 patients (89.8%) were treated appropriately according to the protocol; 31 participants in the control group did not follow the complete protocol.

**Table 1.** Baseline Patient Characteristics in the Full Analysis Set Population

Characteristic	No. (%)			
	Full analysis set		Randomization set	
	Argatroban (n = 302)	Control (n = 307)	Argatroban (n = 314)	Control (n = 314)
Age, median (IQR), y	66 (57-74)	66 (56-74)	66 (57-74)	66 (56-74)
Sex				
Male	191 (63.2)	195 (63.5)	199 (63.4)	201 (64.0)
Female	111 (36.8)	112 (36.5)	115 (36.6)	113 (36.0)
Currently smokes tobacco	100 (33.1)	81 (26.4)	103 (32.8)	83 (26.4)
Comorbidities				
Hypertension	218 (72.2)	208 (67.8)	227 (72.3)	212 (67.5)
Diabetes	83 (27.5)	89 (29.0)	86 (27.4)	91 (29.0)
Prior ischemic or hemorrhagic stroke	38 (12.6)	35 (11.4)	38 (12.1)	36 (11.5)
Hyperlipidemia	33 (10.9)	35 (11.4)	33 (10.5)	35 (11.1)
Coronary heart disease	4 (1.3)	7 (2.3)	4 (1.3)	7 (2.2)
Body mass index, median (IQR)^a^	23.4 (21.2-25.2)	24.0 (22.0-26.0)	23.4 (21.2-25.2)	24.0 (22.0-26.0)
Blood pressure at randomization				
Systolic, median (IQR), mm Hg	150 (140-167)	150 (136-168)	150 (140-167)	151 (136-168)
Diastolic, median (IQR), mm Hg	84 (76-92)	85 (77-94)	84 (76-92)	84 (77-93)
Estimated premorbid function (mRS score)				
No symptoms (0)	285 (94.4)	291 (94.8)	297 (94.6)	298 (94.9)
Symptoms without any disability (1)	17 (5.6)	16 (5.2)	17 (5.4)	16 (5.1)
NIHSS score at randomization, median (IQR)	8 (6-10)	8 (5-10)	8 (6-10)	8 (5-10)
NIHSS score before deterioration, median (IQR)	4 (2-6)	3 (2-6)	4 (2-6)	3 (2-6)
Time from neurological worsening to randomization, median (IQR), h	6 (2-14)	6 (2-13)	6 (2-14)	6 (2-14)
Time from symptom onset to randomization, median (IQR), h	24 (15-34)	23 (13-32)	24 (15-34)	23 (13-32)
Antiplatelet therapy throughout procedure				
Monotherapy	237 (78.5)	202 (65.8)	242 (77.1)	208 (66.2)
Dual antiplatelet therapy	65 (21.5)	105 (34.2)	72 (22.9)	106 (33.8)
Reperfusion treatment	55 (18.2)	64 (20.8)	57 (18.2)	64 (20.4)
Intravenous thrombolysis treatment	53 (17.5)	62 (20.2)	55 (17.5)	62 (19.7)
Endovascular treatment	3 (1.0)	4 (1.3)	3 (1.0)	4 (1.3)
TOAST	(n = 293)	(n = 290)	(n = 303)	(n = 297)
LAA	132 (45.0)	139 (47.9)	135 (44.5)	140 (47.1)
SVO	123 (42.0)	120 (41.4)	129 (42.6)	124 (41.8)
CE	9 (3.1)	8 (2.8)	9 (3.0)	8 (2.7)
SOE	8 (2.7)	4 (1.4)	8 (2.6)	4 (1.3)
SUE	21 (7.2)	19 (6.6)	22 (7.3)	21 (7.1)
Large vessel occlusion	4 (1.3)	9 (2.9)	4 (1.3)	9 (2.9)

Abbreviations: CE, cardioembolism; LAA, large-artery atherosclerosis; mRS, modified Rankin Scale; NIHSS, National Institutes of Health Stroke Scale; SOE, stroke of other determined etiology; SUE, stroke of undetermined etiology; SVO, small-vessel occlusion; TOAST, Trial of Org 10172 in Acute Stroke Treatment.

^a^ Calculated as weight in kilograms divided by height in meters squared.

### Primary Outcome

The percentage of patients with mRS scores of 0 to 3 at 90 days was 80.5% (240 of 298) in the argatroban group and 73.3% (222 of 303) in the control group. In the full analysis set population, the risk of having a good outcome showed a significant difference between the argatroban and control groups (unadjusted RD, 7.2%; 95% CI, 0.6%-14.0%; RR, 1.10; 95% CI, 1.01-1.20; *P* = .04) (Table 2). The difference in the risks of having a good outcome remained significant after adjustment for prespecified prognostic variables (RD, 7.4%; 95% CI, 0.7%-14.1%; RR, 1.10; 95% CI, 1.01-1.20; *P* = .03) (Table 2). The per-protocol analysis yielded similar results (unadjusted RD, 7.3%; 95% CI, 0.4%-14.2%; RR, 1.10; 95% CI, 1.01-1.20; *P* = .04; adjusted RD, 7.2%; 95% CI, 0.4%-14.1%; RR, 1.10; 95% CI, 1.00-1.20; *P* = .04) (eTable 2 in Supplement 3).

**Table 2.** Primary Analysis of Outcomes in the Full Analysis Set

Outcome	No./total No. (%)		Unadjusted			Adjusted^a^		
	Argatroban (n = 302)	Control (n = 307)	Risk difference (95% CI)	Risk ratio (95% CI)	*P* value	Risk difference (95% CI)	Risk ratio (95% CI)	*P* value
Primary								
mRS score of 0 to 3 at 90 d^b^^,^^c^	240/298 (80.5)	222/303 (73.3)	7.2 (0.6 to 14.0)	1.10 (1.01 to 1.20)	.04	7.1 (0.3 to 13.9)	1.10 (1.00 to 1.20)	.04
Secondary								
mRS score of 0 to 2 at 90 d	166/298 (55.7)	152/303 (50.2)	5.5 (−2.4 to 13.5)	1.11 (0.96 to 1.29)	.18	5.1 (−2.9 to 13.1)	1.10 (0.95 to 1.29)	.21
NIHSS score at 90 d, median (IQR)^c^^,^^d^	2 (0 to 4)	2 (0 to 4)	MD: −0.74 (−2.64 to 1.15)		.44	AMD: −0.84 (−2.75 to 1.07)		.39
Barthel scale score at 90 d, median (IQR)	90 (70 to 100)	90 (55 to 100)	MD: 4.69 (−0.50 to 9.86)		.08	AMD: 4.55 (−0.59 to 9.68)		.08
mRS score distribution at 90 d^e^	2 (1 to 3)	2 (1 to 4)	OR: 0.70 (0.53 to 0.93)		.01	OR: 0.71 (0.53 to 0.94)		.02
Stroke or other vascular events within 90 d	17/298 (5.7)	21/303 (6.9)	−1.2 (−5.1 to 2.7)	0.823 (0.44 to 1.53)	.54	−1.2 (−5.1 to 2.8)	0.831 (0.436 to 1.561)	.57

Abbreviations: AMD, adjusted mean difference; MD, mean difference; mRS, modified Rankin Scale; NIHSS, National Institutes of Health Stroke Scale.

^a^ Adjusted for prespecified prognostic variables (age, sex, NIHSS score at randomization, time from the onset of symptoms to randomization).

^b^ mRS Scores range from 0 to 6. A score of 0 indicates no symptoms; 1, symptoms without clinical significant disability; 2, slight disability; 3, moderate disability; 4, moderately severe disability; 5, severe disability; and 6, death.

^c^ Calculated using a generalized linear model.

^d^ NIHSS scores range from 0 to 42, with higher scores indicating greater stroke severity. It is analyzed using a generalized linear model.

^e^ As a post hoc analysis, this outcome was used to describe a shift in measures of functioning according to the full range of scores on the mRS at 90 days.

### Secondary Outcomes

**Figure 2.**
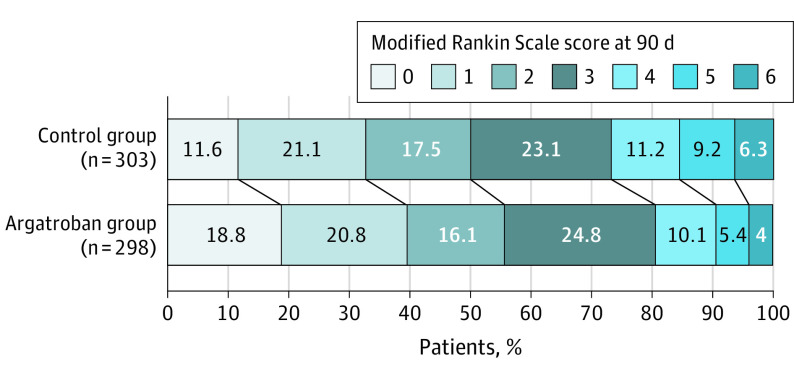
Modified Rankin Scale Score for the Argatroban vs Control Group

Significant differences were observed in the secondary outcomes of the mRS score distribution at 90 days in both the unadjusted and adjusted analysis (Figure 2). No significant differences were observed, including the rate of having an mRS score of 0 to 2, NIHSS score at 90 days, Barthel scale score at 90 days, and stroke or other vascular events within 90 days in the secondary outcomes in both the unadjusted and adjusted analysis (Table 2). In the per-protocol analysis, similar results were obtained in both unadjusted and adjusted analyses (eTable 2 in Supplement 3).

A prespecified subgroup analysis demonstrated a potentially stronger effect of argatroban among female patients (argatroban vs control: female, 81.7% [89 of 109] vs 65.5% [72 of 110]; male, 79.9% [151 of 189] vs 77.7% [150 of 193]; interaction *P* = 0.046) (eTable 3 in Supplement 3). Other subgroup analysis showed characteristics, such as age, NIHSS score at randomization, reperfusion therapy, and time from the onset of symptom to randomization, did not have a significant impact on the effectiveness of the intervention of argatroban in reducing the risks of the primary outcome.

We further conducted a subgroup analysis on patients receiving dual vs mono antiplatelet therapy. There was no significant interaction between subgroups receiving argatroban and different antiplatelet therapies (eTable 3 in Supplement 3).

### Adverse Events

The occurrence of adverse events was similar across the 2 groups, including symptomatic intracranial hemorrhage, parenchymal hematoma, other bleeding events, and other common adverse events (Table 3). The proportion of symptomatic intracranial hemorrhage was 3 of 317 (0.9%) in the argatroban group and 2 of 272 (0.7%) in the control group (*P* = .78).

**Table 3.** Adverse Events in the Safety Set

Adverse event	No./total No. (%)		
	Argatroban (n = 331)	Control (n = 278)	*P* value
Symptomatic intracranial hemorrhage^a^	3/317 (0.9)	2/272 (0.7)	.78
Parenchymal hematoma^b^	1/317 (0.3)	2/272 (0.7)	.46
Other bleeding events^c^	2/331 (0.6)	1/278 (0.4)	.55
Other common adverse events^d^	2/331 (0.6)	0	.15

^a^ The European Cooperative Acute Stroke Study defines symptomatic intracranial hemorrhage as any evidence of bleeding on the head computed tomography imaging associated with clinically significant neurologic deterioration (increase in National Institutes of Health Stroke Scale score ≥4 points) in the opinion of the clinical investigator or independent safety monitor.

^b^ Parenchymal hematoma was defined as confluent bleeding occupying more than 30% of the infarct volume with mass effect.

^c^ Other bleeding events included skin, mucous membrane, gastrointestinal, respiratory tract, urine, and gum bleeding.

^d^ Other most common adverse events included dizziness and vomiting.

## Discussion

This randomized clinical trial shows that the combination of argatroban and antiplatelet therapy resulted in a significantly greater likelihood of good functional outcome at 90 days in patients with END after AIS, with no additional risk of major intracranial or extracranial hemorrhage.

To date and to the authors’ knowledge, the present study was the largest randomized clinical trial to provide robust statistical evidence on the effect of argatroban on END. Early combination of argatroban and antiplatelet use was associated with neurological improvement at 3 months. A subgroup of retrospective study showed that argatroban therapy tended toward superiority over antiplatelet therapy by decreasing stroke severity at discharge, although the difference was not significant in patients with AIS.^9^ Argatroban plus dual antiplatelet therapy yield a significant decrease in the NIHSS score of patients with acute minor posterior circulation ischemic stroke during hospitalization, but the benefit disappeared after propensity score matching.^10^ However, these studies were not designed to assess treatment effects in patients with stroke in progression. Our study was novel in that we targeted a population with END in the hyperacute stroke and showed the benefit of argatroban combination with antiplatelet therapy in reducing the probability of disability.

The use of anticoagulation therapy in AIS has engendered much controversy. Prospective outcome studies of low-molecular-weight heparin in AIS, including the large Trial of Org 10172 in Acute Stroke Treatment (TOAST) study, failed to show any difference in functional outcome in the treated group compared with placebo.^11,12,13^ Patients allocated to heparin in the International Stroke Trial (IST) had significantly fewer recurrent ischemic strokes within 14 days, but this was offset by a similar-sized increase in hemorrhagic strokes; therefore, the difference in death or nonfatal recurrent stroke was not significant.^14^ Due to the excessive hemorrhage transformation rates associated with conventional anticoagulants, most efforts to reduce END have focused on novel anticoagulants combined with antiplatelet therapies. No harmful profile of argatroban was observed even in patients who received intravenous alteplase, suggesting the possible safety of anticoagulants.^15^ In our study, a combined regimen of antiplatelet and argatroban in patients with END did not increase the risk of symptomatic intracerebral hemorrhage and severe systemic bleeding, which is in line with previous studies.^5,15,16^

Recently, a study^17^ found that unexplained END occurring after thrombolysis was independently associated with susceptibility vessel sign extension on magnetic resonance imaging, suggesting that unexplained END may be associated with thrombus extension. We propose that thrombus extension might be due to activation of physiological coagulation cascade because of blood stasis adjacent to the original thrombus. Qazi and coinvestigators recently reported that patients with poor baseline collaterals had longer clots than those with intermediate or good collaterals.^18^ Argatroban works by interfering with the clotting process, making it more difficult for clots to form. Anticoagulation before detection of thrombi is more effective than its use after thrombus formation.^19^ Thrombin inhibition by argatroban also prevents injury to the vascular endothelium, thus facilitating endogenous plasminogen activator production,^20^ making it more difficult for clots to form. Hence, early use of argatroban after END may prevent further thrombus extension and thereby improve the functional outcome. A 7-day administration of combination treatment is sufficient to cover the END high-risk time frame (48-72 hours),^21^ and early intervention produces a better outcome than delayed intervention.^22^ Future studies are required to confirm the ideal timing of anticoagulation therapy after END.

Furthermore, subgroup analyses suggested that early argatroban use was more effective in female patients than male patients. Future research on therapy approaches for END might consider its interaction with sex. Our study also revealed that argatroban treatment appeared to be more advantageous in cases with small-vessel occlusion although without statistical significance. Prior studies have reported hemodynamic effect and excitotoxicity may contribute to the progression of lacunar stroke^23^; thus, there may be targeted mechanisms beyond thrombus propagation from the combination of argatroban and antiplatelet therapy. Future large studies may help to clarify these mechanisms.

In our study, patients randomized to the control group were more likely to get dual antiplatelet therapy, potentially due to the limited drug options for progressive stroke, although no significant effect was found on outcome. Even with more application of dual antiplatelet therapy, the control group had less good functional outcome than the argatroban group, which may indicate the potential effect of argatroban. However, our study was neither powered nor specifically targeted at this treatment effect, and the results should therefore be interpreted with great caution.

### Limitations

This study has several limitations. First, there were more crossover patients in the control group than in the argatroban group, primarily due to the open-label design, which may have biased the study; although, the per-protocol analysis yielded similar results to the full analysis. Second, the current investigation did not conceal the allocated treatment from the participants and physicians because of the open-label design. Nevertheless, blinded–end point assessments were used to mitigate measurement bias and guarantee objective measurement of the primary end point. Third, further confirmation of these conclusions in non-Chinese populations would be welcome, considering the variations in comorbidities and etiology among patients with AIS.

## Conclusions

Results of this randomized clinical trial showed that among patients with AIS and END, treatment with argatroban and antiplatelet agents resulted in a significantly greater likelihood of good functional outcome at 90 days. Trial results provide evidence to support the use of argatroban in patients with END.

## Notes

Supplement 1.
Trial Protocol.

Supplement 2.
Statistical Analysis Plan.

Supplement 3. **eTable 1.** Baseline Patient Characteristics in the Per-Protocol Set Population **eTable 2.** Primary Analysis of Outcomes in the Per-Protocol Set **eTable 3.** Primary Outcome by Subgroups in the Full Analysis Set

Supplement 4.
Data Sharing Statement.
