# Supplementary material for: Argatroban in Patients With Acute Ischemic Stroke With Early Neurological Deterioration: A Randomized Clinical Trial
Source: JAMA Neurol. 2024 Jan 8;81(2):118–25. doi: 10.1001/jamaneurol.2023.5093 (PMC10775075; doi:10.1001/jamaneurol.2023.5093)
Supplement: Supplement 1. — Trial Protocol. [file jamaneurol-e235093-s001.pdf]

PROTOCOL

Version 2.0 of 11/21/2019

Efficacy of Argatroban in acute ischemic Stroke with Early neurological deterioration (EASE): a prospective, randomised, open-lable, blinded-end point, multi-centre trial

Principle Investigator:
Prof Min Lou
Department of Neurology
The 2nd affiliated hospital of Zhejiang University
Hangzhou, 310000, China
Tel: 86-13958007213
E-mail: lm99@zju.edu.cn

The protocol was drafted before the trial began, and was amended to Version 2.0 (2019-11-21).

Table of Contents

1. Background and current state of knowledge 7
2. Objectives of the trial 7
    2.1 Hypothesis tested 7
    2.2 Primary objective 7
    2.3 Secondary objectives 7
3. Design and selection of patients 7
    3.1 Trail plan 7
    3.2 Selection criteria: 8
    3.3 Duration of participation for each subject 8
    3.4 Randomization and measures to reduce bias 9
4. Plan and conduct of the trial 9
    4.1 Clinical trial flow chart 9
    4.2 Study completion 10
    4.3 Study termination 10
5. Study medications 10
    5.1 Identification of study drugs 10
    5.2 Responsibilities of study drugs administration 10
6. Treatment administered to included subjects 10
    6.1 Description of the treatment required to conduct the study 10
    6.2 Permitted and prohibited medical drugs and treatments in the study 10
7. Outcome measurements 10
    7.1 Primary efficacy outcome 10
    7.2 Secondary efficacy outcomes 10
    7.3 Safety outcomes 11
    7.4 Study flow diagram 11
    7.5 Study periods 11
8. Description of safety assessment parameters 11
    8.1 Evaluate clinical safety 11
    8.2 Safety assessment 12
    8.3 Safety report 13
9. Statistics 14
    9.1 Sample size 14
    9.2 Statistical analysis plan 15
    9.3 Analysis population 15
10. Data management and monitoring 15
    10.1 Training of study site personnel 15
    10.2 Monitoring of the study 15

Abstract

**Title**	Efficacy of Argatroban in acute ischemic Stroke with Early neurological deterioration (EASE): a prospective, randomized, open-label, blinded-end point, multi-centre trial
**Principle Centre**	The 2nd affiliated hospital of Zhejiang University
**Objective**	To explore the efficacy and safety of Argatroban for ischemia stroke with early neurological deterioration
**Efficacy Outcome**	Primary outcome:<br>1. Proportion of modified Rankin Score (mRS, 0-3) at 90±3 days after randomization<br>Secondary outcome：<br>1. Proportion of modified Rankin Score (mRS, 0-2) at 90±3 days after randomization；<br>2. Recovery assessed by categorical shift in mRS at 90 days;<br>3. National Institutes of Health stroke scale (NIHSS) score at 7 days and 90±3 days after randomization;<br>4. The rate of composite cardiovascular events at 90±3 days, including cerebrovascular events, myocardial infarction, angina pectoris and systemic embolism.<br>5. Barthel scale score at 90±3 days.
**Safety Outcome**	1. Proportion of symptomatic intracerebral haemorrhage;<br>2. Proportion of parenchymal hematoma type 2;<br>3. Other most common adverse events.
**Trial Design**	This is a prospective, randomized, open-label, blinded-endpoint, multicentre trial. Subjects included are randomly assigned into two groups: the experiment group and the control group. Follow-up was to be performed at baseline, 24 hours, 7±1 days, 30±3 days, and 90±3 days after randomization. The primary outcome assessors were masked to the allocation assignment.
**Trial Population**	Patients with acute ischemic stroke developed early neurological deterioration
**Sample Size**	628
**Inclusion criteria**	1. Age >18 years;<br>2. Patients presented with clinical signs of acute ischemic stroke with 48 hours of stroke onset (for stroke with unknown time of onset, the midpoint of the time last known to be well and symptom recognition time);<br>3. Early neurological deterioration with National Institutes of Health Stroke Scale score ≥2 increase within 48 hours after stroke onset;<br>4. Informed consent from patient, family member or legally responsible person depending on local ethics requirements.
**Exclusion criteria**	1. Cardiogenic cerebral embolism;<br>2. Intracranial hemorrhage or lesions larger than two thirds of the territory

	of the middle cerebral artery shown on CT;<br>3. Pre-stroke mRS score of > 1;<br>4. Contraindication for argatraban;<br>5. Treat with tirofiban;<br>6. Severe heart, liver or kidney dysfunction, defined as left ventricular ejection fraction < 40%; glutamic-pyruvic transaminase or glutamic oxalacetic transaminase increased to three times of the upper limit of normal; creatinine clearance < 30ml/min;<br>7. Severe disease with a life expectancy of less than 3 months;<br>8. Any condition that could impose hazards to the patient if study therapy is initiated or affect the participation of the patient in the study (this applies to patients with severe microangiopathy such as hemolytic uremic syndrome or thrombotic thrombocytopenic purpura). The judgment is left to the discretion of the investigator.
**Trial Cycle**	All included patients were followed up at baseline, 24 hours, 7±1 days, 30±3 days, and 90±3 days after randomization, respectively.
**Treatment Regimens**	Eligible patients were randomly (a ratio of 1:1) assigned into the experimental group: argatroban (Argatroban treatment was as follows: continuous infusion with a dose of 60 mg/d for 2 days, followed by 20mg per day for 5 days), or the control group: standard therapy according to Chinese guidelines for diagnosis and treatment of acute ischemic stroke 2018.
**Procedure**	**Screening period**: On day 0 (baseline period), it is necessary to complete enrolment screening, and collect demographic characteristics, medical history (including history of hypertension, diabetes and drug treatment history, etc), brain imaging (computer tomography or magnetic resonance imaging), neurological measurements (NIHSS score, mRS score), haematological examination (blood routine, blood glucose, hepatic and renal function, coagulation routine, etc) and other information.<br>**Treatment period**: The patients received argatroban plus standard therapy or standard therapy only.<br>**Follow-up period**: NIHSS score was assessed at 24 hours, 7±1 days, and 90±3 days after randomization. The mRS score was assessed at 90±3 days after randomization. All concomitant medications, adverse events, stroke recurrence and other vascular events of each visit were recorded since the last visit.<br>All the adverse events of included subjects should be recorded and tracked until properly resolved.<br>All the serious adverse events of included subjects should be recorded and tracked, even if the subjects have finished the trial, until the events were resolved, or stabilization judged by the investigator.
**Concomitant Treatment**	Guideline-based treatment
**Statistical Analysis**	Intention-to-treat analysis will be used to compare the treatment effect

	between two groups and all the data will be analysed with Software Statistical Product and Service Solutions (version 23). The mean standard deviation will be used if the continuous data were normally distributed, and the median and interquartile range will be used if the continuous data were non-normally distributed.categorical data will be expressed as number (percentage). when comparing the data of two groups, t test or Mann-Whitney test was used for continuous data, and chi-square test was used for categorical data.<br>Logistic regression analysis will be performed for the proportion of a good outcome at 90 days, a favourable outcome at 90 days, NIHSS score at 90 days, Barthel score at 90 days, composite cardiovascular events at 90±3 days and safety endpoints. An ordinal regression analysis will be conducted to investigate a treatment effect across the mRS scale. Generalised linear model was used to compare NIHSS score and barthel score at 90 days. Statistical tests were considered significant when the two-sided P value was less than 0.05.
**Sites Number**	About 30
**Duration**	24-36months

Abbreviation

Abbreviation	Full title
AE	Adverse Event
AIS	Acute Ischemic Stroke
ALT	Alanine Aminotransferase
AST	Aspartate Transaminase
APTT	Activated Partial Thromboplastin Time
BUN	Blood Urea Nitrogen
Cr	Creatinine
CRF	Clinical Research Form
CT	Computed Tomography
GCP	Good Clinical Practice
GLU	Glucose
IQR	Inter-Quartile Range
ITT	Intention to Treat
MRI	Magnetic Resonance Imaging
mRS	Modified Rankin Scale
NIHSS	National Institute of Health Stroke Scale
PPS	Per Protocol Set
PT	Prothrombin Time
rtPA	Recombinant tissue-type plasminogen activator
SAE	Serious Adverse Event
SD	Standard Deviation
SS	Safety Set
TC	Total Cholesterol
TG	Triglyceride
TOAST	Trial of Org 10 172 in acute stroke treatment

1. Background and current state of knowledge

Currently, most patients with acute ischemic stroke, whether they receive IV-rtPA or not, are admitted to acute stroke units for prevention and monitoring of early complications. While a sizeable proportion of patients exhibit signs of improvement in this early post-stroke period, another significant proportion shows no improvement, or even worse experience deterioration in the severity of the neurological deficit, so-called early neurological deterioration (END). END results in poor outcome in stroke, despite the use of antiplatelet and thrombolytic therapies[1]. Therefore, it is necessary to explore an effective and simple way to achieve improvement in acute ischemic stroke patients with END.

Argatroban is a selective thrombin inhibitor which can directly inhibit free and clot-associated thrombin and thrombin-induced activities. Clinical studies have shown that argatroban is safe and may offer benefits in patients with AIS. Herein, this study aims to investigate the safety and efficacy of argatroban in improving the functional outcome without increasing the risk of intracerebral haemorrhage in END patients.

2. Objectives of the trial

2.1 Hypothesis tested

This study intends to demonstrate that the argatroban plus standard therapy will be superior to standard therapy alone in improving functional outcomes in AIS patients with END.

2.2 Primary objective

To test the hypothesis that the argatroban plus standard therapy will be superior to standard therapy alone in improving good functional outcomes in AIS patients with END.

2.3 Secondary objectives

1. To determine the proportion of favourable functional outcome at 90 days by treatment group.
2. Recovery assessed by categorical shift in mRS at 90 days;
3. National Institutes of Health stroke scale (NIHSS) score at 7 days and 90±3 days after randomization;
4. The rate of composite cardiovascular events at 90±3 days, including cerebrovascular events, myocardial infarction, angina pectoris and systemic embolism.
5. Barthel scale score at 90±3 days.

3. Design and selection of patients

3.1 Trail plan

This is a prospective, randomized, open-label, blinded-endpoint, multicentre trial.

The patients were randomly assigned into two groups:

Experimental group: intravenous argatroban (Argatroban treatment was as follows: continuous infusion with a dose of 60 mg/d for 2 days after randomization, followed by 20mg per day for 5 days).

Control group: standard therapy according to Chinese guidelines for diagnosis and treatment of acute ischemic stroke 2018.

Both groups of patients were given standard guideline-based treatment until to 90 days.

Follow-up was performed at baseline, 24 hours, 7±1 days, and 90±3 days after randomization,

respectively.

The primary outcome will be measured without knowledge of the allocation assignment by trained investigators in each trail site.

3.2 Selection criteria

Inclusion criteria: in order to be eligible, the patients must meet all the following criteria:

1) Age >18 years;

2) Patients presented with clinical signs of acute ischemic stroke with 48 hours of stroke onset (for stroke with unknown time of onset, the midpoint of the time last known to be well and symptom recognition time);

3) Early neurological deterioration with National Institutes of Health Stroke Scale score ≥2 increase within 48 hours after stroke onset;

4) Informed consent from patient, family member or legally responsible person depending on local ethics requirements.

Exclusion criteria: in order to be included the patients must not have any of the following criteria:

1) Cardiogenic cerebral embolism, patients with any definite source of cardiac embolism were excluded from the trial: chronic or paroxysmal atrial fibrillation, sick sinus syndrome, mitral stenosis, mechanical valve, endocarditis, intracardiac clot or vegetation, myocardial infarction within three months, dilated cardiomyopathy, left atrial spontaneous echo contrast, ejection fraction less than 30%. There will be no retrospective exclusion of patients based on information coming to knowledge later on.

2) Intracranial hemorrhage or lesions larger than two thirds of the territory of the middle cerebral artery shown on CT;

3) Pre-stroke mRS score of > 1;

4) Contraindication for argatraban;

5) Current treat with tirofiban;

6) Severe heart, liver or kidney dysfunction, defined as left ventricular ejection fraction < 40%; glutamic-pyruvic transaminase or glutamic oxalacetic transaminase increased to three times of the upper limit of normal; creatinine clearance < 30ml/min;

7) Severe disease with a life expectancy of less than 3 months;

8) Any condition that could impose hazards to the patient if study therapy is initiated or affect the participation of the patient in the study (this applies to patients with severe microangiopathy such as hemolytic uremic syndrome or thrombotic thrombocytopenic purpura). The judgment is left to the discretion of the investigator.

Suspension criteria

In order to protect the interests and rights of subjects, guarantee the quality of the trial and avoid unnecessary economic losses, we set out trial suspension criteria as follows:

1. The proportion of serious adverse events during the trial is higher than 10%, or an independent Data Monitoring Committee (DMC) terminate the study unconditionally.
2. One treatment is found to be significantly better than the other.

3.3 Duration of participation for each subject

Each subject will be followed up in the trial for 3 months.

3.4 Randomization and measures to reduce bias

Patients are randomly assigned to either treatment or control arms using a secure, web-based randomization system. The randomization scheme is the combination of minimization and the biased coin method and is never deterministic. A dynamic stratification system will ensure well-balanced subgroups. The randomization algorithm will employ biased-coin minimization and the variance method with stratification weights. The strategy is to balance treatment assignment along the marginal distribution of each stratification factor. The stratification factors used and their hierarchy will be: 1) age, 2) sex, and 3) NIHSS at randomization. Study outcomes assessors are blinded to treatment assignment. 90-day mRS was evaluated through a structured interview for telephone assessment. We held a training course for all investigators at each centre to ensure validity and reproducibility.

4. Plan and conduct of the trial

4.1 Clinical trial flow chart

Item / Period	Screening	Follow-up	
Visit	1	2	3
Time	0 day	7±1 days	90±3 days
Inclusion/Exclusion Criteria	×		
Sign informed consent	×		
Randomization	×		
Suspension criteria		×	×
Demographic characteristics	×		
Medical history	×		
Physical examination	×	×	×
Brain CT/MRI	×	×	
ECG (12 lead)	×		
NIHSS score	×	×	×
mRS score	×		×
Blood routine	×		
Coagulation routine	×		
Blood biochemistry	×		
Concomitant medication	×	×	×

Item \ Period	Screening	Follow-up	
Adverse events	×	×	×
Stroke recurrence and other vascular events		×	×

4.2 Study completion

The study is considered to be finished when the last visit of the last subject in the trial is completed.

4.3 Study termination

The sponsor retains the right to close the study centre or suspend the study at any time. The study centre should be closed after the study is completed. The centre is considered closed after all required documentation and study supplies have been collected and a centre closure visit has been conducted.

The sponsor or investigator can close the centre in advance according to this reason:

Investigator fails to comply with the requirements of the study protocol, IEC/IRB or local regulatory authorities, sponsor's operating procedures or GCP guidelines.

5. Study medications

5.1 Identification of study drugs

Argatorban is manufactured by Tianjin Institute of Pharmaceutical Research Co., Ltd.

5.2 Responsibilities of study drugs administration

The investigator of the clinical trial is responsible for ensuring the study drugs during the whole process of the study.

6. Treatment administered to included subjects

6.1 Description of the treatment required to conduct the study

The experimental group received intravenous argatroban (Argatroban treatment was as follows: continuous infusion with a dose of 60 mg/d for 2 days, followed by 20mg per day for 5 days). Argatroban infusion was terminated immediately if major systemic bleeding or symptomatic intracerebral hemorrhage was suspected. The control group received standard therapy according to Chinese guidelines for diagnosis and treatment of acute ischemic stroke 2018.

6.2 Permitted and prohibited medical drugs and treatments in the study

Tirofiban is prohibited for this study.

7. Outcome measurements

7.1 Primary efficacy outcome

Proportion of modified Rankin Score (mRS, 0-3) at 90±3 days after randomization

7.2 Secondary efficacy outcomes

1. Proportion of modified Rankin Score (mRS, 0-2) at 90±3 days after randomization;

2. Recovery assessed by categorical shift in mRS at 90 days;

3. National Institutes of Health stroke scale (NIHSS) score at 7 days and 90±3 days after randomization;

4. The rate of composite cardiovascular events at 90±3 days, including cerebrovascular events, myocardial infarction, angina pectoris and systemic embolism.

5. Barthel scale score at 90±3 days.

7.3 Safety outcomes

1. Proportion of symptomatic intracerebral haemorrhage;
2. Proportion of parenchymal hematoma type 2;
3. Other most common adverse events.

7.4 Study flow diagram

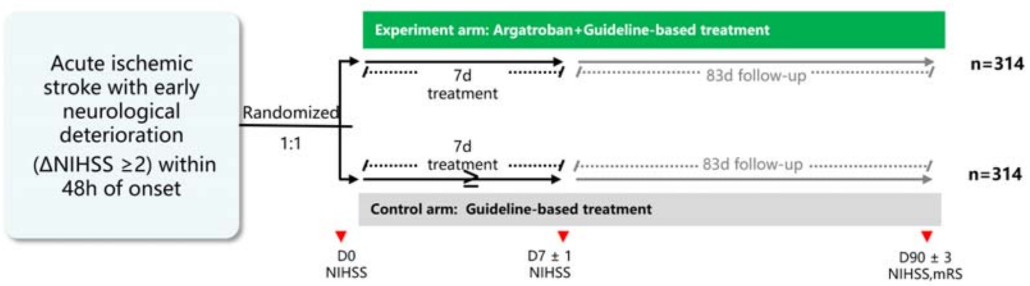

7.5 Study periods

Screening period: On day 0 (baseline period), it is necessary to complete enrolment screening, and collect demographic characteristics, medical history (including history of hypertension, diabetes, atrial fibrillation and drug treatment history, etc), brain imaging (CT or MRI), neurological measurements (NIHSS score, mRS score), haematological examination (blood routine, blood glucose, hepatic and renal function, coagulation, electrocardiogram, etc) and other information. The index event, the END and the enrollment had to occur within 48 hours. Treatment period: The two groups were given intravenous agatroban (experimental group) or standard therapy (control group). All the patients were given standard guideline-based treatment. Brain CT or MRI were performed at 7±1 days after randomization.

Follow-up period: NIHSS score was assessed at 7±1 days, and 90±3 days after randomization. The mRS score was assessed at 90±3 days after randomization. All concomitant medications, adverse events, stroke recurrence and other vascular events of each visit were recorded since the last visit.

All the adverse events of included subjects should be recorded and tracked until properly resolved.

All the serious adverse events of included subjects should be recorded and tracked, even if the subjects have finished the trial, until the events were resolved, or stabilization judged by the investigator.

8. Description of safety assessment parameters

8.1 Evaluate clinical safety

Physical examinations, including neurological assessments, imaging studies were performed

at baseline, 48 hours, 7±1 days, and 90±3 days (if necessary) after randomization; adverse event of each visit was collected after baseline visit.

Adverse events monitoring

All information on adverse events, whether mentioned by subjects, discovered by investigators, or discovered through physical examination, laboratory tests, and other methods, should be recorded on the adverse events page of the case report form and handled in accordance with appropriate regulations and report.

Adverse Event (AE)

The adverse event is any adverse medical event that occurs in a study subject during a study that is not necessarily related to this treatment. It is included that any events that are new or that have worsened in severity and frequency from baseline, including abnormal results from diagnostic methods such as laboratory test.

Serious Adverse Event (AE)

Serious adverse event is any adverse medical event at any dose that meets one or more of the following criteria：

Cause death

Life-threatening

Cause significant or permanent disability or impairment of organ function

Significant medical event or need for intervention

Causing or prolonging hospitalisation

8.2 Safety assessment

For add adverse events in clinical studies, the following factors must be evaluated for safety:

Severity criteria for SAE

The causal relationship between the event and the investigational drug

Severity of incident

Anticipation of events

Causal relationship between events and the study drugs

Regardless of serious adverse events or non-serious adverse events, the investigator must evaluate the relevance of the event to use the study drugs according to the following criteria:

Evaluating association between adverse events and study treatments

5-level classification	Judgment criteria
Definitely related	The time of initiation of use is reasonably related to the time of occurrence of the adverse event; the adverse event corresponds to the known adverse reaction type of the study treatment; the occurrence of the adverse event cannot be explained by factors other than the study treatment (such as concomitant medication); the adverse events reduce or disappear after study treatment dose reduction or discontinuation; similar adverse events (non-essential) can occur with re-use of study treatment

Probably related	The time of initiation of use is reasonably related to the time of occurrence of the adverse event; the adverse event corresponds to the known adverse reaction type of the study treatment; the occurrence of the adverse event can or cannot be explained by factors other than the study treatment (such as concomitant medication); the adverse event can or cannot reduce or disappear after study treatment dose reduction or discontinuation; it is not sure whether similar adverse events (non-essential) can occur with re-use of study treatment
Probably unrelated	The time of initiation of use is not reasonably related to the time of occurrence of the adverse event; the adverse event corresponds to the known adverse reaction type of the study treatment; the occurrence of the adverse event can or cannot be explained by factors other than the study treatment (such as concomitant medication); the adverse event can or cannot reduce or disappear after study treatment dose reduction or discontinuation; it is not sure whether similar adverse events (non-essential) can occur with re-use of study treatment
Definitely unrelated	The time of initiation of use is not reasonably related to the time of occurrence of the adverse event; the adverse event corresponds to the known adverse reaction type of the study treatment; the occurrence of the adverse event can or cannot be explained by factors other than the study treatment (such as concomitant medication); the adverse event can or cannot reduce or disappear after study treatment dose reduction or discontinuation; similar adverse events (non-essential) cannot occur with re-use of study treatment.
Unjudged	The judgment cannot be made due to incomplete, contradictory information or the fact that the information cannot be supplemented and verified

8.3 Safety report

All AEs, regardless of their severity, nature of severity, or their causal relationship to study treatment, from the time of signed informed consent until the last follow-up visit (90 days after first use of the drugs), are to be recorded in the original and recorded in medical terms on the clinical research form (CRF). When symptoms and signs are caused by common causes, a diagnosis of the disease should be given where possible (e.g., cough, runny nose, sneezing, sore throat, and headache should be reported as "upper respiratory tract infection"). The investigator must make a judgment on the causal relationship between the AE and the study treatment and record it on the clinical research form. All treatments for AEs should be documented in the original medical record and reported as requested by the sponsor.

All SAEs in clinical research must be reported to the ethics committee of the centre, the principal investigator of the centre, the sponsor, the contract research organization, the research team leader unit, and the adverse reaction monitoring centre within 24 hours; Report to the ethics committee and data and safety monitoring committee of the team leader unit within 7 natural days after being informed.

Investigators must complete a SAE form to record the time, severity, duration, actions taken and outcomes of SAEs. Any follow-up information for SAEs should also be reported in writing within 24 hours according to the above process.

All SAE that do not resolve at study termination or subject early withdrawal must be followed up to any of the following:

1. Incident mitigation
2. Events are stable
3. If the baseline value is known, the event returns to the baseline value
4. Events can be attributed to other drugs or factors not related to study
5. It is unlikely that any further information will be available (subject or physician refuses to provide further information and remains lost to follow-up after various follow-up attempts)

Abnormal laboratory test results

During the study, when the results of laboratory tests meet the following conditions, they must be regarded as AEs and recorded in medical terms on the AEs of the CRF:

1. Accompanying clinical symptoms
2. Causing a change in the trial protocol (e.g., treatment interruption or discontinuation)
3. lead to a change in medical intervention or concomitant treatment
4. Clinically significant as judged by the investigator (medical and scientific methods should be used to judge whether an isolated laboratory abnormality is an AE)

When the AE meets the serious criteria, it should be reported in accordance with the SAE reporting procedure.

9. Statistics

9.1 Sample size

The sample size was estimated according to the results of the previous observational cohort (with the same inclusion and exclusion criteria as the RCT) at the study center, 33 patients in the experimental group and 66 patients in the control group. There were no significant differences in age and NIHSS score (baseline and progressive time), and the proportion of mRS 0-3 at Day 90 in

the experimental group and the control group was 80% and 69.6% , respectively. Based on 0.8 power to detect a significant difference (p=0.05, two-sided), and to compensate for non-evaluable patients of 15%, a total sample size of up to 628 patients would be required with about 314 in each of treatment and placebo arms.

9.2 Statistical analysis plan

Intention-to-treat (ITT) analysis will be used to analyze the therapeutic effect of the two groups and all the data will be analyzed with SPSS 23.0 Software. The mean standard deviation (SD) will be used if the data are normally distributed, and the median and quartile spacing (IQR) will be used if the data are non-normally distributed. Count data are expressed as n(%).When comparing the data of two samples, t test or rank sum test was used for measurement data, and chi-square test was used for count data. Multivariate analysis was performed using multiple logistic regression and general linear models. There is statistical significance if $P<0.025$ (one-side test).

9.3 Analysis population

Intention-To-Treat (ITT)

The ITT population includes all subjects who were randomly assigned to a treatment group on an intention-to-treat basis, including subjects who planned to receive treatment but did not actually receive it.

Per Protocol Set (PPS)

The protocol-compliant set is a subset of the FAS, which will include completion of all treatments or at least the determination of the primary end point as required by the research protocol, and good compliance (the ratio of the actual dose to the applied dose is between 80% and 120%). Subjects with no serious protocol violation (the definition of serious protocol violation will be specified in the Statistical Analysis Plan) constitute the PPS analysis set for this study.

Safety Set (SS)

The SS analysis population included all subjects who received at least one study protocol treatment and had at least one safety evaluation.

10. Data management and monitoring

10.1 Training of study site personnel

The sponsor will organize a training in each site before the first patients is entered. The training will cover all the staff related to the study, such as medical, nursing and other staff. Instructions including the trial protocol, investigator’s brochure, scale evaluation, using electronic data capture system, SAE report procedure and so on.

10.2 Monitoring of the study

A stuff from sponsor will have regular contacts with study sits and will be available whenever the investigator needs information about the study. Regular visits includes as follows:

●Confirm the adherence to the protocol and timely recorded data in the CRFs

●Verify data validity, including timely informed consent, clinical data, laboratory results, neuroimaging data, and neurological function evaluation (NIHSS, mRS) at baseline and follow-up

Appendix

Appendix 1: National Institutes of Health Stroke Scale (NIHSS)

Instructions	Scale Definition	Score
**1a. Level of Consciousness:** The investigator must choose a response if a full evaluation is prevented by such obstacles as an endotracheal tube, language barrier, orotracheal trauma/bandages. A 3 is scored only if the patient makes no movement (other than reflexive posturing) in response to noxious stimulation.	0 = **Alert;** keenly responsive.<br>1 = **Not alert**; but arousable by minor stimulation to obey, answer, or respond.<br>2 = **Not alert**; requires repeated stimulation to attend, or is obtunded and requires strong or painful stimulation to make movements (not stereotyped).<br>3 = Responds only with reflex motor or autonomic effects or totally unresponsive, flaccid, and flexic.	______
**1b. LOC Questions:** The patient is asked the month and his/her age. The answer must be correct - there is no partial credit for being close. Aphasic and stuporous patients who do not comprehend the questions will score 2. Patients unable to speak because of endotracheal intubation, orotracheal trauma, severe dysarthria from any cause, language barrier, or any other problem not secondary to aphasia are given a 1. It is important that only the initial answer be graded and that the examiner not "help" the patient with verbal or non-verbal cues.	0 = **Answers** both questions correctly.<br>1 = **Answers** one question correctly.<br>2 = **Answers** neither question correctly.	______
**1c. LOC Commands:** The patient is asked to open and close the eyes and then to grip and release the non-paretic hand. Substitute another one step command if the hands cannot be used. Credit is given if an unequivocal attempt is made but not completed due to weakness. If the patient does not respond to command, the task should be demonstrated to him or her (pantomime), and the result scored (i.e., follows none, one or two commands). Patients with trauma, amputation, or other physical impediments should be given suitable one-step commands. Only the first attempt is scored.	0 = **Performs** both tasks correctly.<br>1 = **Performs** one task correctly.<br>2 = **Performs** neither task correctly.	______
**2. Best Gaze:** Only horizontal eye movements will be tested. Voluntary or reflexive (oculocephalic) eye movements will be scored, but caloric testing is not done. If the patient has a conjugate deviation of the eyes that can be overcome by voluntary or reflexive activity, the score will be 1. If a patient has an isolated peripheral nerve paresis (CN III, IV or VI), score a 1. Gaze is testable in all aphasic patients. Patients with ocular trauma, bandages, pre-existing blindness, or other disorder of visual acuity or fields should be tested with reflexive movements, and a choice made by the investigator. Establishing eye contact and then moving about the patient from side to side will occasionally clarify the presence of a partial gaze palsy.	0 = **Normal.**<br>1 = **Partial gaze palsy;** gaze is abnormal in one or both eyes, but forced deviation or total gaze paresis is not present.<br>2 = **Forced deviation,** or total gaze paresis not overcome by the oculocephalic maneuver.	______

Instructions	Scale Definition	Score
**3. Visual:** Visual fields (upper and lower quadrants) are tested by confrontation, using finger counting or visual threat, as appropriate. Patients may be encouraged, but if they look at the side of the moving fingers appropriately, this can be scored as normal. If there is unilateral blindness or enucleation, visual fields in the remaining eye are scored. Score 1 only if a clear-cut asymmetry, including quadrantanopia, is found. If patient is blind from any cause, score 3. Double simultaneous stimulation is performed at this point. If there is extinction, patient receives a 1, and the results are used to respond to item 11.	**0 = No visual loss.**<br>**1 = Partial hemianopia.**<br>**2 = Complete hemianopia.**<br>**3 = Bilateral hemianopia** (blind including cortical blindness).	____
**4. Facial Palsy**: Ask – or use pantomime to encourage – the patient to show teeth or raise eyebrows and close eyes. Score symmetry of grimace in response to noxious stimuli in the poorly responsive or non-comprehending patient. If facial trauma/bandages, orotracheal tube, tape or other physical barriers obscure the face, these should be removed to the extent possible.	**0 = Normal** symmetrical movements.<br>**1 = Minor paralysis** (flattened nasolabial fold, asymmetry on smiling).<br>**2 = Partial paralysis** (total or near-total paralysis of lower face).<br>**3 = Complete paralysis** of one or both sides (absence of facial movement in the upper and lower face).	____
**5. Motor Arm:** The limb is placed in the appropriate position: extend the arms (palms down) 90 degrees (if sitting) or 45 degrees (if supine). Drift is scored if the arm falls before 10 seconds. The aphasic patient is encouraged using urgency in the voice and pantomime, but not noxious stimulation. Each limb is tested in turn, beginning with the non-paretic arm. Only in the case of amputation or joint fusion at the shoulder, the examiner should record the score as untestable (UN), and clearly write the explanation for this choice.	**0 = No drift**; limb holds 90 (or 45) degrees for full 10 seconds.<br>**1 = Drift;** limb holds 90 (or 45) degrees, but drifts down before full 10 seconds; does not hit bed or other support.<br>**2 = Some effort against gravity**; limb cannot get to or maintain (if cued) 90 (or 45) degrees, drifts down to bed, but has some effort against gravity.<br>**3 = No effort against gravity**; limb falls.<br>**4 = No movement.**<br>**UN** = Amputation or joint fusion, explain: ____________<br><br>**5a. Left Arm**<br><br>**5b. Right Arm**	____<br>____

Instructions	Scale Definition	Score
**6. Motor Leg:** The limb is placed in the appropriate position: hold the leg at 30 degrees (always tested supine). Drift is scored if the leg falls before 5 seconds. The aphasic patient is encouraged using urgency in the voice and pantomime, but not noxious stimulation. Each limb is tested in turn, beginning with the non-paretic leg. Only in the case of amputation or joint fusion at the hip, the examiner should record the score as untestable (UN), and clearly write the explanation for this choice.	**0 = No drift;** leg holds 30-degree position for full 5 seconds.<br>**1 = Drift;** leg falls by the end of the 5-second period but does not hit bed.<br>**2 = Some effort against gravity;** leg falls to bed by 5 seconds, but has some effort against gravity.<br>**3 = No effort against gravity;** leg falls to bed immediately.<br>**4 = No movement.**<br>**UN** = Amputation or joint fusion, explain: ________________<br>**6a. Left Leg**<br>**6b. Right Leg**	_____<br>_____
**7. Limb Ataxia:** This item is aimed at finding evidence of a unilateral cerebellar lesion. Test with eyes open. In case of visual defect, ensure testing is done in intact visual field. The finger-nose-finger and heel-shin tests are performed on both sides, and ataxia is scored only if present out of proportion to weakness. Ataxia is absent in the patient who cannot understand or is paralyzed. Only in the case of amputation or joint fusion, the examiner should record the score as untestable (UN), and clearly write the explanation for this choice. In case of blindness, test by having the patient touch nose from extended arm position.	**0 = Absent.**<br>**1 = Present in one limb.**<br>**2 = Present in two limbs.**<br>**UN = Amputation or joint fusion**, explain: ______________________________	_____
**8. Sensory:** Sensation or grimace to pinprick when tested, or withdrawal from noxious stimulus in the obtunded or aphasic patient. Only sensory loss attributed to stroke is scored as abnormal and the examiner should test as many body areas (arms [not hands], legs, trunk, face) as needed to accurately check for hemisensory loss. A score of 2, "severe or total sensory loss," should only be given when a severe or total loss of sensation can be clearly demonstrated. Stuporous and aphasic patients will, therefore, probably score 1 or 0. The patient with brainstem stroke who has bilateral loss of sensation is scored 2. If the patient does not respond and is quadriplegic, score 2. Patients in a coma (item 1a=3) are automatically given a 2 on this item.	**0 = Normal;** no sensory loss.<br>**1 = Mild-to-moderate sensory loss**; patient feels pinprick is less sharp or is dull on the affected side; or there is a loss of superficial pain with pinprick, but patient is aware of being touched.<br>**2 = Severe to total sensory loss;** patient is not aware of being touched in the face, arm, and leg.	_____

Instructions	Scale Definition	Score
**9. Best Language:** A great deal of information about comprehension will be obtained during the preceding sections of the examination. For this scale item, the patient is asked to describe what is happening in the attached picture, to name the items on the attached naming sheet and to read from the attached list of sentences. Comprehension is judged from responses here, as well as to all of the commands in the preceding general neurological exam. If visual loss interferes with the tests, ask the patient to identify objects placed in the hand, repeat, and produce speech. The intubated patient should be asked to write. The patient in a coma (item 1a=3) will automatically score 3 on this item. The examiner must choose a score for the patient with stupor or limited cooperation, but a score of 3 should be used only if the patient is mute and follows no one-step commands.	**0 = No aphasia;** normal.<br>**1 = Mild-to-moderate aphasia;** some obvious loss of fluency or facility of comprehension, without significant limitation on ideas expressed or form of expression. Reduction of speech and/or comprehension, however, makes conversation about provided materials difficult or impossible. For example, in conversation about provided materials, examiner can identify picture or naming card content from patient's response.<br>**2 = Severe aphasia**; all communication is through fragmentary expression; great need for inference, questioning, and guessing by the listener. Range of information that can be exchanged is limited; listener carries burden of communication. Examiner cannot identify materials provided from patient response.<br>**3 = Mute**, global aphasia; no usable speech or auditory comprehension.	____
**10. Dysarthria:** If patient is thought to be normal, an adequate sample of speech must be obtained by asking patient to read or repeat words from the attached list. If the patient has severe aphasia, the clarity of articulation of spontaneous speech can be rated. Only if the patient is intubated or has other physical barriers to producing speech, the examiner should record the score as untestable (UN), and clearly write an explanation for this choice. Do not tell the patient why he or she is being tested.	**0 = Normal.**<br>**1 = Mild-to-moderate dysarthria**; patient slurs at least some words and, at worst, can be understood with some difficulty.<br>**2 = Severe dysarthria;** patient's speech is so slurred as to be unintelligible in the absence of or out of proportion to any dysphasia, or is mute/anarthric.<br>UN = **Intubated** or other physical barrier, explain:___________________________ ___	____
**11. Extinction and Inattention (formerly Neglect):** Sufficient information to identify neglect may be obtained during the prior testing. If the patient has a severe visual loss preventing visual double simultaneous stimulation, and the cutaneous stimuli are normal, the score is normal. If the patient has aphasia but does appear to attend to both sides, the score is normal. The presence of visual spatial neglect or anosagnosia may also be taken as evidence of abnormality. Since the abnormality is scored only if present, the item is never untestable.	**0 = No abnormality.**<br>**1 = Visual, tactile, auditory, spatial, or personal inattention** or extinction to bilateral simultaneous stimulation in one of the sensory modalities.<br>**2 = Profound hemi-inattention or extinction to more than one modality;** does not recognize own hand or orients to only one side of space.	____

Instructions	Scale Definition	Score
	**Total NIHSS:**	____

Appendix 2: Modified Rankin Score (mRS)

Grade	Description
0	No symptoms
1	Symptoms without any incapacity (able to perform all usual activities)
2	Mild incapacity (unable to perform all usual activities but able to look after his/her affairs alone)
3	Moderate incapacity (requires assistance but walks alone)
4	Severe incapacity (requires assistance for walking and physical body needs)
5	Severe incapacity (bedbound, incontinent, permanent surveillance required)
6	Death

Reference

1. Park MG, Oh EH, Kim BK, Park KP. Intravenous tissue plasminogen activator in acute branch atheromatous disease: does it prevent early neurological deterioration? J Clin Neurosci. 2016;33:194–197. doi: 10.1016/j.jocn.
