# Supplementary material for: Argatroban in Patients With Acute Ischemic Stroke With Early Neurological Deterioration: A Randomized Clinical Trial
Source: JAMA Neurol. 2024 Jan 8;81(2):118–25. doi: 10.1001/jamaneurol.2023.5093 (PMC10775075; doi:10.1001/jamaneurol.2023.5093)
Supplement: Supplement 2. — Statistical Analysis Plan. [file jamaneurol-e235093-s002.pdf]

Efficacy of Argatroban in acute ischemic Stroke with Early neurological deterioration (EASE): a prospective, randomised, open-label, blinded-end point, multi-centre trial

Statistical analysis plan

ClnicalTrials.gov registration number: NCT04275180

Protocol version and date: Version 1.0, 09/27/2019

Chief Investigator: Prof Min Lou

Trial statistician: Changzheng Yuan

SAP authors: Xuting Zhang, Wansi Zhong， Min Lou

**SAP version history**		
Version Date	SAP Version#	Details of Changes
09/27/2019	1.0	First version

Table of contents

1. Introduction 5
    1.1 Purpose of the statistical analysis plan 5
    1.2 Background to the study 5
2. Study objectives and outcomes 5
    2.1 Study objectives 5
    2.1.1 Primary Objective 5
    2.1.2 Secondary Objectives 5
    2.2 Outcomes 6
    2.2.1 Primary outcome 6
    2.2.2 Secondary outcomes 6
3. Study design 6
    3.1 Design 6
    3.2 Trial sites 6
    3.3 Treatments 7
    3.4 Randomisation 7
    3.5 Sample size 7
4. Analysis populations 7
    4.1 Study population data sets 7
    4.2 Analysis close date 8
    4.3 Data cleaning 8
    4.4 Data download 8
5. Statistical analyses 8
    5.1 Primary outcome analysis 8
        5.1.1 mTT analysis of the primary outcome - the primary analysis 8
        5.1.2 PP analysis of the primary outcome 9
        5.1.3 Covariate adjusted analysis of the primary outcome 9
        5.1.4 Subgroup analysis of the primary outcome 9
    5.2 Secondary outcome analysis 9
    5.3 Exploratory analysis 9
6. Safety analyses 9
    6.1 Safety variables 9
7. General considerations for data analyses 10
    7.1 Covariates analyses 10
    7.2 Subgroup analysis 10
    7.3 Multiplicity 10
    7.4 Missing data 10
    7.5 Further exploratory analyses 10
    7.6 Data summaries 10
8. References 11

Abbreviation

Abbreviation	Full title
AE	Adverse Event
AIS	Acute Ischemic Stroke
ALT	Alanine Aminotransferase
AST	Aspartate Transaminase
APTT	Activated Partial Thromboplastin Time
BUN	Blood Urea Nitrogen
Cr	Creatinine
CRF	Clinical Research Form
CT	Computed Tomography
GCP	Good Clinical Practice
GLU	Glucose
IQR	Inter-Quartile Range
ITT	Intention to Treat
MRI	Magnetic Resonance Imaging
mRS	Modified Rankin Scale
NIHSS	National Institute of Health Stroke Scale
PPS	Per Protocol Set
PT	Prothrombin Time
rtPA	Recombinant tissue-type plasminogen activator
SAE	Serious Adverse Event
SD	Standard Deviation
SS	Safety Set
TC	Total Cholesterol
TG	Triglyceride
TOAST	Trial of Org 10 172 in acute stroke treatment

1. Introduction

1.1 Purpose of the statistical analysis plan

In this statistical analysis plan (SAP), we delineate the outcome variables, statistical methods, and analysis strategies in order to make comparison between the argatroban with guideline-based treatment in acute ischemic stroke with early neurological deterioration.

1.2 Background to the study

Currently, most patients with acute ischemic stroke, whether they receive intravenous recombinant tissue plasminogen activator or not, are admitted to acute stroke units for prevention and monitoring of early complications. While a sizeable proportion of patients exhibit signs of improvement in this early post-stroke period, another significant proportion shows no improvement, or even worse experience deterioration in the severity of the neurological deficit, so-called early neurological deterioration. Early neurological deterioration (END) results in poor outcome in stroke, despite the use of antiplatelet and thrombolytic therapies[1]. How to improve the neurological function of END patients has been brought to the fore by data as the rapid rise of neuroprotective agents in animal experiments. By now, no acknowledged treatment was recommended by the guidelines.

Therefore, it is necessary to explore an effective way to achieve improvement in acute ischemic stroke patients with END. Argatroban is a selective thrombin inhibitor which can directly inhibit free and clot-associated thrombin and thrombin-induced activities. Clinical studies have shown that argatroban is safe and may offer benefits in patients with AIS. There is growing evidence from research which demonstrated the promise of argatroban therapy in improving the prognosis after stroke.

Herein, this study aims to investigate the safety and efficacy of argatroban in improving the functional outcome without increasing the risk of intracerebral haemorrhage in END patients.

2. Study objectives and outcomes

2.1 Study objectives

2.1.1 Primary Objective

To test the hypothesis that the argatroban plus standard therapy will be superior to standard therapy alone in improving good functional outcomes in AIS patients with END.

2.1.2 Secondary Objectives

1. To determine the proportion of favourable functional outcome at 90 days by treatment group.
2. To determine the distribution of mRS at 90 days by treatment group;
3. To determine National Institutes of Health stroke scale (NIHSS) score at 7 days and 90±3 days after randomisation by treatment group;
4. To determine the rate of composite cardiovascular events at 90±3 days, including cerebrovascular events, myocardial infarction, angina pectoris and systemic embolism by treatment group.
5. To determine the Barthel scale score at 90±3 days.

2.2 Outcomes

2.2.1 Primary outcome

The primary outcome is the occurrence of mRS (0-3) at 90 days (binary outcome), characterized by a score of 0-3 on the mRS for assessment of neurological disability at 90 days after randomisation through telephone.

2.2.2 Secondary outcomes

1) The proportion of favourable functional outcome at 90 days by treatment group.
2) Recovery assessed by categorical shift in mRS at 90 days;
3) National Institutes of Health stroke scale (NIHSS) score at 7 days and 90±3 days after randomisation;
4) The rate of composite cardiovascular events at 90±3 days, including cerebrovascular events, myocardial infarction, angina pectoris and systemic embolism.
5) Barthel scale score at 90±3 days.

3. Study design

3.1 Design

This is a prospective, randomised, open-label, blinded-endpoint, multicentre trial.

3.2 Trial sites

Twenty-eight hospitals will participate this clinical trial. Based on previous good cooperation on clinical trials of acute ischemic stroke, these trial sites were selected to reveal a spectrum of China health care institutions.

Department of Neurology, the 2nd Affiliated Hospital of Zhejiang University, Hangzhou, China
Department of Neurology, The Second Affiliated Hospital of Jiaxing University, Jiaxing, China
Department of Neurology, Haiyan People's Hospital, Jiaxing, China
Department of Neurology, First hospital of Ninghai county, Ningbo, China
Department of Neurology, People's hospital of Anji, Huzhou, China
Department of Neurology, Yiwu Central Hospital, Yiwu, China
Department of Neurology, The Affiliated People's Hospital of Ningbo University, Ningbo, China
Department of Neurology, Putuo Hospital, Zhoushan, China
Department of Neurology, Dongyang Affiliated Hospital of Wenzhou Medical University, Dongyang, China
Department of Neurology, Zhuji People’s Hospital, Zhuji, China
Department of Neurology, Quzhou traditional Chinese medicine hospital, Quzhou, China
Department of Neurology, Xiangshan People’s Hospital, Xiangshan, China
Department of Neurology, Institute of Geriatric Neurology,The Second Affiliated Hospital and Yuying Children’s Hospital, Wenzhou Medical University, Wenzhou, China
Department of Neurology, Shaoxing Second Hospital, Shaoxing, China
Department of Neurology, The Affiliated Hospital of Hangzhou Normal University, Hangzhou, China
Department of Neurology, The Second People's Hospital of Yuhang District, Hangzhou, China
Department of Neurology, The affiliated hospital of Medicine School, Ningbo University, Ningbo, China

Department of Neurology, Ningbo Ninth Hospital, Ningbo, China
Department of Neurology, Ningbo Medical Center Lihuili Hospital, Ningbo, China
Department of Neurology, Quzhou City kecheng district people's hospital, Quzhou, China
Department of Neurology, Zhoushan Hospital, Wenzhou Medical University, Zhoushan, China
Department of Neurology, Affiliated Hospital of Jiaxing University, Jiaxing, China
Department of Neurology, Tongxiang Hospital of Traditional Chinese Medicine, Jiaxing, China
Department of Neurology, Zhenhai Longsai hospital of Ningbo city, Ningbo, China
Department of Neurology, Zhejiang University, Hangzhou, China
Department of Neurology, The 4th affiliated hospital of Zhejiang University, School of Medicine, Yiwu, China
Department of Neurology, Ningbo No.2 Hospital, Ningbo, China
Department of Neurology, Ningbo Hangzhou Hospital, Hangzhou, China

3.3 Treatments

Experimental group: intravenous argatroban (Argatroban treatment was as follows: continuous infusion with a dose of 60 mg/d for 2 days after randomisation, followed by 20mg per day for 5 days).

Control group: standard therapy according to Chinese guidelines for diagnosis and treatment of acute ischemic stroke 2018.

Both groups of patients were given standard guideline-based treatment until to 90 days.

3.4 Randomisation

Patients are randomly assigned to either treatment or control arms using a secure, web-based randomization system. The randomization scheme is the combination of minimization and the biased coin method and is never deterministic. A dynamic stratification system will ensure well-balanced subgroups. The randomization algorithm will employ biased-coin minimization and the variance method with stratification weights. The strategy is to balance treatment assignment along the marginal distribution of each stratification factor. The stratification factors used and their hierarchy will be: 1) age, 2) sex, and 3) NIHSS at randomization.

3.5 Sample size

The sample size was estimated according to the results of the previous observational cohort (with the same inclusion and exclusion criteria as the RCT) at the study center, 33 patients in the experimental group and 66 patients in the control group. There were no significant differences in age and NIHSS score (baseline and progressive time), and the proportion of mRS 0-3 at Day 90 in the experimental group and the control group was 80% and 69.6% , respectively. Based on 0.8 power to detect a significant difference (p=0.05, two-sided), and to compensate for non-evaluable patients of 15%, a total sample size of up to 628 patients would be required with about 314 in each of treatment and placebo arms.

4. Analysis populations

4.1 Study population data sets

Before the database lock, each analysis set and the reasons for exclusion will be determined and documented. When the subjects have protocol deviation/violation, the reason will be listed in a

summary table. Two study populations will be studied in the analysis to determine efficacy and safety, as follows:

(1) Intention-To-Treat (ITT)

The ITT population includes all subjects who were randomly assigned to a treatment group on an intention-to-treat basis, including subjects who planned to receive treatment but did not actually receive it.

(2) Per Protocol Set (PPS)

The PPS population include completion of all treatments or at least the determination of the primary end point as required by the research protocol, and good compliance (the ratio of the actual dose to the applied dose is between 80% and 120%). Subjects with no serious protocol violation (the definition of serious protocol violation will be specified in the Statistical Analysis Plan) constitute the PPS analysis set for this study.

(3) Safety Set (SS)

The SS analysis population included all subjects who received at least one study protocol treatment and had at least one safety evaluation.

4.2 Analysis close date

The analysis close date is defined as the date on which the last participant completed 90-day follow-up.

Last contact date, which is also shown as Trial reference end date, is the date on which the trial procedure last related. For survival subjects it is defined as the maximum of:

Date of last office visit (scheduled or unscheduled visit)

Date of the last follow-up contact (including last date on subject survival status recorded)

Date of the last known adverse event (AE) status or lab results reported on the AE or lab case report form (CRF) pages, respectively.

4.3 Data cleaning

Missing data will be properly coded. Researchers will review the data to ensure there are no erroneous entries.

4.4 Data download

The database will be locked when all data have been loaded and reviewed. The data will be downloaded into SPSS formats for statistical analyses.

5. Statistical analyses

The analyses will be performed by the trial statistician and the primary analysis will be checked by a second statistician. This study adopt the ITT analyses as the main strategy analysis for the Primary and secondary outcomes.

5.1 Primary outcome analysis

5.1.1 ITT analysis of the primary outcome - the primary analysis

The primary outcome is good functional outcome defined as mRS (0-3) at 90 days, which is a binary outcome. The primary endpoint will be based on the ITT population as described above.

The primary endpoint will be summarized by number (%) of participants that have good functional outcome by treatment group. A generalized linear models will be performed, in which

the occurrence of good functional outcome at 90 days is the response variable and the treatment is the only predictor. This model will derive relative risk (RR) and risk difference (RD) of achieving the primary outcome between the Argartroban group and Control group with p value and two-sided 95% confidence interval (CI).

5.1.2 PP analysis of the primary outcome

We will also carry out an analysis of the primary outcome on the PP population, using the same statistical methods in Section 5.1.1.

5.1.3 Covariate adjusted analysis of the primary outcome

In order to make sure whether the treatment effect is affected with the inclusion of covariables, we will also perform the adjusted analyses including following covariables:

Age, Sex, NIHSS score at randomization, Time from the onset of symptoms to randomization

This model derive the adjusted odds ratio (OR) and 95% CI comparing the Argartroban group and Control group.

Missing covariates on baseline will be imputed as the following description in 8.5 missing data.

5.1.4 Subgroup analysis of the primary outcome

These followed covariates by age (<65 years or ≥years), sex (female or male), NIHSS (≤8 or >8), time from onset to treatment (≤24hours or ＞24hours), and whether receiving reperfusion therapy will be included in subgroup analyses by carrying out the above analysis separately for each category of a subgroup covariate.

Homogeneity of treatment effect by a subgroup variable was evaluated through a binary logistic regression, in which the treatment, subgroup variable and their interaction term are predictors. P value will be displayed for the interaction term.

5.2 Secondary outcome analysis

Secondary outcome analyses will be based on the ITT and PP populations.

Proportion of mRS (0-2) at 90 days and the occurrence of stroke or other vascular events within 90d will be treated as a binary outcome and will be calculated by number (%) of participants with event by treatment group and analysed by the same analysis as the primary endpoint. This model derive the adjusted RR, RD and 95% CI comparing the Argartroban group and Control group.

5.3 Exploratory analysis

If necessary, we will use other statistical methods which is defined as exploratory.

6. Safety analyses

6.1 Safety variables

Adverse events (AEs) will be restricted to those events happened within the 90 days after randomization. Adverse events was described as the number of AEs, the number (%) of participants with AEs by the Argatroban group.

Safety analyses will focus on the number of any adverse medical events and serious adverse events (SAEs) occurring after randomisation.

Summaries of the total number of reported AEs/SAEs and number of participants reporting at least

one AE/SAE will be presented by treatment received and overall. Moreover, summaries of the suspected relationship with trial treatment, suspected trial treatment or other cause, duration of recovered SAEs, seriousness criteria, event outcome, DAIDS grade and SAE, will be presented by treatment received and overall.

Line listings of all reported SAEs for each participant will also be presented by treatment received. They will include (where appropriate):

Randomised treatment
DAIDS grade
Event description
Seriousness criteria
Suspected relationship to the trial medications
Suspected products
Other causality
Expectedness
Date of randomisation
Date of onset
Date event became serious (serious events only)
Date of recovery
Outcome
Details of the treatment received

7. General considerations for data analyses

All data analyses will be done by SPSS (version 23).

7.1 Covariates analyses

Covariate analyses will be performed on the primary outcome and secondary outcomes on the ITT and PP populations.

7.2 Subgroup analysis

Subgroup analyses will be performed for the primary outcome on the ITT and PP populations. Homogeneity of treatment effect by a subgroup variable was evaluated through a binary logistic regression, in which the treatment, subgroup variable and their interaction term are predictors. P value will be displayed for the interaction term.

7.3 Multiplicity

Secondary outcomes and additional analyses for the primary outcome are exploratory in nature, thus, we will not apply multiplicity adjustment.

7.4 Missing data

Simple imputation methods will be used in the covariate adjusted analysis for missing baseline covariates. If it is a continuous variable, missing values will be imputed with average number calculated from the available sample. If missing variable is categorical, we will impute it using the most frequent value from the sample.

7.5 Further exploratory analyses

If deemed to be needed, exploratory analyses will be added to this analysis plan with reasonable explanation.

7.6 Data summaries

Continuous variables will be summarised according to number of subjects with non-missing data (n), mean, standard deviation (SD), median, minimum, and maximum.
Categorical variables will be summarised according to the absolute frequency and percentage of participants (%) in each category level. The denominator for the percentages is the number of subjects in the treatment arm with data available.

8. References

1) Park MG, Oh EH, Kim BK, Park KP. Intravenous tissue plasminogen activator in acute branch atheromatous disease: does it prevent early neurological deterioration? J Clin Neurosci. 2016;33:194–197. doi: 10.1016/j.jocn.
