# Supplementary material for: Argatroban in Patients With Acute Ischemic Stroke With Early Neurological Deterioration: A Randomized Clinical Trial
Source: JAMA Neurol. 2024 Jan 8;81(2):118–25. doi: 10.1001/jamaneurol.2023.5093 (PMC10775075; doi:10.1001/jamaneurol.2023.5093)
Supplement: Supplement 3. — eTable 1. Baseline Patient Characteristics in the Per-Protocol Set Population eTable 2. Primary Analysis of Outcomes in the Per-Protocol Set eTable 3. Primary Outcome by Subgroups in the Full Analysis Set [file jamaneurol-e235093-s003.pdf]

Supplementary Online Content

Zhang X, Zhong W, Xue R, et al. Argatroban in patients with acute ischemic stroke with early neurological deterioration: a randomized clinical trial. *JAMA Neurol*. Published online January 8, 2024. doi:10.1001/jamaneurol.2023.5093

eTable 1. Baseline Patient Characteristics in the Per-Protocol Set Population

eTable 2. Primary Analysis of Outcomes in the Per-Protocol Set

eTable 3. Primary Outcome by Subgroups in the Full Analysis Set

This supplementary material has been provided by the authors to give readers additional information about their work.

© 2024 Zhang X et al. *JAMA Neurology.*

eTable 1 Baseline patient characteristics in the per- protocol set population

Characteristic	No.(%) Per protocol set Argatroban (n=292)	Control (n=272)
Age, median (IQR), y	66 (57,74)	66 (56,74)
Men	185 (63.4)	173 (63.6)
Currently smokes tobacco	99 (33.9)	73 (26.8)
Comorbidities		
Hypertension	212 (72.6)	182 (66.9)
Diabetes	82 (28.1)	77 (28.3)
Prior ischemic or hemorrhagic stroke	37 (12.7)	31 (11.4)
Hyperlipidemia	33 (10.9)	28 (10.3)
Coronary heart disease	4 (1.4)	7 (2.6)
Body mass index, median (IQR)	23.4 (21.0, 25.2)	24.1 (22.0, 26.1)
Blood pressure at randomization		
Systolic		
Median (IQR), mmHg	150 (140,167)	150 (136, 168)
>140mmHg		
Diastolic		
Median (IQR), mmHg	84 (76,92)	84 (77, 94)
>90mmHg		
Estimated premorbid function (mRS score)		
No symptoms (score of 0)	275 (94.2)	258 (94.6)
Symptoms without any disability (score of 1)	17 (5.8)	14 (5.1)
NIHSS score at randomization, median (IQR)	8 (6,10)	8 (5,10)
NIHSS score before deterioration, median (IQR)	4 (2, 6)	3 (2, 6)
Time from symptom onset to randomization, median (IQR), h	24 (15,34)	23 (12,31)
Antiplatelet therapy throughout procedure		
Monotherapy	230 (78.8)	184 (67.6)
Dual antiplatelet therapy	62 (21.2)	88 (32.4)
Reperfusion treatment	53 (18.2)	58 (21.3)
Intravenous thrombolysis treatment	51 (17.5)	56 (20.6)
Endovascular treatment	3 (1.0)	4 (1.5)

© 2024 Zhang X et al. *JAMA Neurology.*

eTable 2 . Primary Analysis of Outcomes in the per-protocol Set

Outcome	No.(%)		Unadjusted			Adjusted[a]		
	Argatroban (n=292)	Control (n=272)	Risk difference (95% CI)	Risk ratio (95% CI)	P value	Risk difference (95% CI)	Risk ratio (95% CI)	P value
**Primary**								
mRS score of 0 to 3 at 90d[b,c]	236/292(80.8%)	200/272(73.5%)	7.3% (0.4% to 14.2%)	1.099 (1.005 to 1.203)	**0.038**	7.1% (0.1% to 14.0%)	1.096 (1.001 to 1.201)	**0.048**
**Secondary**								
mRS score of 0 to 2 at 90d	163/292(55.8%)	138/272(50.7%)	5.1% (-3.2% to 13.3%)	1.100 (0.942 to 1.285)	0.228	4.3% (-3.9% to 12.5%)	1.087 (0.929 to 1.272)	0.299
NIHSS score at 90d, median (IQR)[d]	2 (0, 4)	2 (0, 4)	MD: -0.982 (-2.881 to 0.917)		0.311	AMD: -1.058 (-2.958 to 0.841)		0.275
Barthel scale score at 90d, median (IQR)	90 (70,100)	90 (55,100)	MD: 4.961 (-0.351 to 10.272)		**0.068**	AMD: 4.615 (-0.618 to 9.849)		**0.084**
mRS score distribution at 90d[e]	2 (1-3)	2 (1-4)	OR: 0.707 (0.528 to 0.947)		**0.020**	OR: 0.723 (0.538 to 0.971)		**0.031**
Stroke or other vascular events within 90d	17/292(5.8%)	18/272(6.6%)	-0.8% (-4.8% to 3.2%)	0.879 (0.459 to 1.680)	**0.696**	-0.7% (-4.7% to 3.4%)	0.902 (0.465 to 1.743)	**0.757**

Abbreviations: mRS, modified Rankin Scale; NIHSS, National Institutes of Health Stroke Scale; MD, mean difference; AMD adjusted mean difference

a Adjusted for prespecified prognostic variables (age, sex, NIHSS score at randomization, time from the onset of symptoms to randomization)

b mRS scores range from 0 to 6; a score of 0 indicates no symptoms; 1, symptoms without clinical significant disability; 2, slight disability; 3, moderate disability; 4,

© 2024 Zhang X et al. *JAMA Neurology.*

moderately severe disability; 5, severe disability; and 6, death.
c calculated using a generalized linear model
d NIHSS scores range from 0 to 42, with higher scores indicating greater stroke severity. It is analyzed using a generalized linear model.
e as a post hoc analysis, this outcome was used to describe a shift in measures of functioning according to the full range of scores on the mRS at 90 days.

© 2024 Zhang X et al. *JAMA Neurology.*

eTable 3 Primary Outcome by Subgroups in the Full Analysis Set

Subgroups	Patients No.	Argatroban group	Control group	Adjusted Risk Ratio (95% CI)	P value	Interaction p value
**Age (years)**						
< 65	282	122/143 (85.3)	110/139 (79.1)	1.068 (0.955 to 1.194)	0.248	0.747
⩾65	319	118/155 (76.1)	112/164 (68.3)	1.110 (0.965 to 1.278)	0.144	
**Sex**						
Female	219	89/109 (81.7)	72/110 (65.5)	1.235 (1.049 to 1.455)	0.012	**0.046**
Male	382	151/189 (79.9)	150/193 (77.7)	1.023 (0.920 to 1.138)	0.671	
**NIHSS score at randomization**						
⩽8	360	143/178 (80.3)	139/182 (76.4)	1.046 (0.936 to 1.169)	0.430	0.104
＞8	241	97/120 (80.8)	83/121 (68.6)	1.170 (1.004 to 1.385)	0.045	
**Reperfusion therapy**						
Yes	118	40/54 (74.1)	46/64 (71.9)	1.005 (0.793 to 1.274)	0.964	0.384
No	483	200/244 (82.0)	176/239(73.6)	1.115 (1.012 to 1.229)	0.029	
**Time from the onset of index event symptoms to randomization (hours)**						
⩽24	328	128/161 (79.5)	130/167 (77.8)	1.025 (0.913 to 1.151)	0.677	0.112
＞24	273	112/137 (81.8)	92/136 (67.6)	1.188 (1.031 to 1.369)	0.018	
**DAPT**						0.412
Yes	167	53/63 (84.1)	74/104 (71.2)	1.169 (0.978-1.394)	0.087	
No	434	187/235 (79.6)	148/199 (74.4)	1.072 (0.967-1.190)	0.188	
**TOAST**						0.662
LAA	268	101/132 (76.5)	98/136 (72.1)	1.065 (0.923-1.228)	0.389	
SVO	241	101/121 (84.3)	90/120 (75.0)	1.128 (0.980-1.026)	0.071	

© 2024 Zhang X et al. *JAMA Neurology.*

Other etiologies	69	31/38 (81.6)	24/31 (77.4)	1.026 (0.792-1.334)	0.845

DAPT, dual antiplatelet therapy; TOAST, Trial of Org 10172 in Acute Stroke Treatment; LAA, large-artery atherosclerosis; SVO, small-vessel occlusion; Other etiologies, including cardioembolism, stroke of other determined etiology and stroke of undetermined etiology.

© 2024 Zhang X et al. *JAMA Neurology.*
