# Supplementary material for: Argatroban in Patients With Acute Ischemic Stroke With Early Neurological Deterioration: A Randomized Clinical Trial
Source: JAMA Neurol. 2024 Jan 8;81(2):118–25. doi: 10.1001/jamaneurol.2023.5093 (PMC10775075; doi:10.1001/jamaneurol.2023.5093)
Supplement: Supplement 4. — Data Sharing Statement. [file jamaneurol-e235093-s004.pdf]

Data Sharing Statement

Zhang. Argatroban in Patients With Acute Ischemic Stroke With Early Neurological Deterioration. *JAMA Neurol*. Published January 08, 2024. doi:10.1001/jamaneurol.2023.5093

Data
Data available: No
